# Revolutionizing Antibody Discovery: An Innovative AI Model for Generating Robust Libraries

**DOI:** 10.1016/j.gpb.2023.06.001

**Published:** 2023-06-22

**Authors:** Yaojun Wang, Shiwei Sun

**Affiliations:** 1 College of Information and Electrical Engineering, China Agricultural University, Beijing 100083, China; 2 Key Laboratory of Intelligent Information Processing, Institute of Computing Technology, Chinese Academy of Sciences, Beijing 100080, China; 3 University of Chinese Academy of Sciences, Beijing 100049, China

## Background

Artificial intelligence models of natural language are becoming increasingly adept at processing and ‘understanding’ language. They are widely used in applications such as automated speech recognition, translation, smart assistants, and text generation [1]. Recently, language models have been applied to such problems as protein function prediction, protein evolution analysis, and protein design [2].

Antibodies are now a crucial therapeutic option for various diseases due to their high specificity and affinity toward antigens. Although antibody libraries hold immense potential in discovering novel treatment targets, their utility is constrained by the significant time and expense required for their generation, as well as numerous sequences with inadequate chemical properties that necessitate re-engineering before they can be employed therapeutically.

In silicon, antibody design is an emergent topic with notable progress, but no existing methods are aimed at solving the multi-property optimization problem in antibody design.

A report in *Genomics, Proteomics & Bioinformatics* by Xu et al. [3] advances the application of antibody design. They utilized a generative pre-trained transformer (GPT) model in combination with reinforcement learning to create innovative antibody sequences. Using this approach, they achieved a remarkable success in generating antibody sequences that possess multiple desirable properties for the third complementarity-determining region of the heavy chain (CDRH3).

## AB-Gen: antibody library design with GPT and deep reinforcement learning

The new tool, AB-Gen [3], developed by Gao Lab (http://cemse.kaust.edu.sa/sfb) at the King Abdullah University of Science and Technology (KAUST), Saudi Arabia, in collaboration with groups in China, is the first-of its kind to solve the multi-property optimization problem in antibody design. The authors constructed an autoregressive model, with the aim of generating new sequences.

Firstly, Xu et al. [3] trained a prior GPT model based on the GPT-2 framework with ∼ 6 million parameters. They used ∼ 75 million CDRH3 sequences from the Observed Antibody Space (OAS) database to represent the entire space of CDRH3 space. About ∼ 10% of the data was left out for model evaluation.

They evaluated the capability of the prior model to learn the CDRH3 space, and analyzed properties including viscosity, clearance, immunogenicity, and sequence similarity distributions of generated samples. The prior and the baseline sequences exhibited similar distributions for these properties. The results indicate that model has learned a good distribution of the CDRH3 space. Additionally, three basic metrics — uniqueness, novelty, and diversity — were calculated to evaluate the generative capability of the models. Together, these results show that the prior model generates CDRH3 sequences with high levels of uniqueness and diversity, and can generate novel sequences not observed in the training dataset. However, these sequences did not have reasonable specificity for human epidermal growth factor receptor 2 (HER2).

Next, Xu et al. [3] adopted a second strategy, reinforcement learning. This was used to fine-tune the GPT model and guide it toward optimizing the generation of CDRH3 sequences with desirable properties. The GPT model was utilized as the policy network for the agent. The reward functions for reinforcement learning were determined based on the likelihoods of CDRH3 sequences and the predicted properties when the entire sequence was sampled. During the reinforcement learning process, the probability of generating CDRH3s with desirable properties increased, while that of generating CDRH3s with unfavorable properties decreased. The prior likelihood was also used to give feedback to the agent to preserve information about the CDRH3 space learned by the prior model.

In this study, two agent models were trained. One agent model, named Agent_HER2, was trained with only HER2 specificity as the scoring function. The other agent model, named Agent_MPO, was trained with multiple property predictors combined as the scoring function. This enabled the fulfillment of multiple requirements in the design of antibody libraries.

The results show that both Agent_HER2 and Agent_MPO models have better HER2 specificity than the prior model. Agent_HER2 generated sequences with higher average HER2 specificity than Agent_MPO, whereas Agent_MPO, which optimized multiple properties, achieved a higher success rate (the ratio of sequences fulfilling multi-property constraints) than Agent_HER2. In short, the results showed that the prior model could learn the sequence space of CDRH3 and generate sequences with similar property distributions as the training dataset. Furthermore, both Agent_HER2 and Agent_MPO were capable of generating novel CDRH3 sequences that met the predefined property constraints, but Agent_MPO achieved a notably higher success rate in generating sequences with desirable properties.

Finally, the authors used AB-Gen to design novel antibody libraries that have the potential for practical antibody discovery. Agent_MPO generated ten thousand sequences, which were filtered using previous property constraints in the success rate calculation in combination with CamSol solubility scores (≥ 0.42, the score for Herceptin). The authors obtained a final set of 509 CDRH3 sequences as the potential library for further analysis.

Some typical properties of these sequences have been analyzed. The maximum edit distance was eight, the minimum was two, and a median edit distance of six was found. As the whole editing range has a length of ten, it can be inferred that approximately 60% of the sequences were modified based on median analysis. This suggests that AB-Gen is capable of designing novel sequences that are not intuitive to create.

The sequence logo for 509 CDRH3 sequences was generated by aligning the sequences using ClustalW. The resulting alignment was then used to create a sequence logo through WebLogo, with the beginning two residues of the CDRH3 (S97 and R98) and the trailing residue Y109 fixed as references. Analysis of the sequence logo revealed that residues G103, Y105, and D108 on the heavy chain of Herceptin are highly conserved among these sequences. This suggests that these protein residues may play essential roles in the binding Herceptin and HER2.

To gain insight into how Herceptin binds to HER2 at the molecular level and potentially explain the functions of the conserved residues, the researchers performed molecular dynamics (MD) simulations for the HER2–Herceptin antigen-binding fragment (Fab) system and examined the molecular interactions in the binding region at the atomic resolution. The MD simulations showed that Herceptin mainly interacts with HER2 through hydrogen bonds.

Specifically, the backbone oxygen atom of residue G103 in Herceptin could form a hydrogen bond with the side chain of residue K593 in HER2, and the side chain of residue D108 in Herceptin is hydrogen bonded with the hydroxyl group of residue Y588 in HER2 (hydrogen bonding probability = 39.5% ± 1.9%). Moreover, the side chain of residue Y105 in Herceptin could form hydrogen bonds with the backbone oxygen atom (31.5% ± 3.2%) and the side chain oxygen atoms (38.7% ± 7.7%) of residue D570 in HER2. These observations not only consolidate the observation from sequence logos that residues G103, Y105, and D108 are essential to bridge HER2 and Herceptin, but also suggest that hydrogen bonds contribute to the interactions between HER2 and Herceptin Fab in the binding region.

As the authors have mentioned, this method can also be used for designing large proteins. To apply this method to other proteins, two primary inputs are required: homologous sequences for pre-training the prior model and a property predictor for providing feedback to the agent. Homologous sequences can be found in sequence databases through homology searches. The primary constraint for applying this method is to calculate scores for desirable properties such as affinity/specificity, activity, solubility, and stability to guide the reinforcement learning framework.

To conclude, Xu et al. [3] have built an AB-Gen tool for designing antibody sequences. The model of the tool uses the network architecture of GPT in combination with a reinforcement learning strategy. Although the GPT model is capable of generating sequences that conform to the distribution space of the target sequence family, it cannot generate sequences with specific properties. Reinforcement learning is used to adjust the direction of the model to conditionally generate sequences. The results indicate that GPT in combination with reinforcement learning framework holds great potential for use in antibody library design, thus empowering the antibody discovery and development process.

On one hand, the proposed method is conceptually similar to ChatGPT, in which the pre-trained language model is combined with the reinforcement learning to generate designs that satisfy the specified property. On the other hand, the proposed model is more advanced than ChatGPT in the sense that it can simultaneously optimize multiple properties, whereas ChatGPT only optimizes one property in the reinforcement learning process. This new method sheds light on the application of ChatGPT and its analogs on protein design, which is, currently, the hottest topic in bioinformatics.

## Competing interests

Both authors have declared no competing interests.

## CRediT authorship contribution statement

**Yaojun Wang:** Writing – original draft. **Shiwei Sun:** Conceptualization, Writing – review & editing. Both authors have read and approved the final manuscript.

## References

[1] Ouyang L., Wu J., Jiang X., Almeida D., Wainwright C.L., Mishkin P., Koyejo S., Mohamed S., Agarwal A., Belgrave D., Cho K., Oh A. (2022). Advances in Neural Information Processing Systems.

[2] Ferruz N., Schmidt S., Höcker B. (2022). ProtGPT2 is a deep unsupervised language model for protein design. Nat Commun.

[3] Xu X., Xu T., Zhou J., Liao X., Zhang R., Wang Y. (2023). AB-Gen: antibody library design with generative pre-trained transformer and deep reinforcement learning. Genomics Proteomics Bioinformatics.

